# Supplementary material for: Assessing the Reliability and Validity of a Questionnaire Evaluating Medical Students’ Attitudes, Knowledge, and Perceptions of Antibiotic Education and Antimicrobial Resistance in University Training
Source: Antibiotics (Basel). 2024 Nov 23;13(12):1126. doi: 10.3390/antibiotics13121126 (PMC11672510; doi:10.3390/antibiotics13121126)
Supplement: Supplementary file 1 [file antibiotics-13-01126-s001.zip › antibiotics-3274123-supplementary.pdf]

Suplemmentary material S1

Table S1. Comparison of total variances explained in different PCAs

Total variance explained											
PCA of original data				Ridge PCA				Bootstrapping PCA			
Component	Initial eigenvalues			Component	Initial eigenvalues			Component	Initial eigenvalues		
	Total	% Explained variance	% Cumulative variance		Total	% Explained variance	% Cumulative variance		Total	% Explained variance	% Cumulative variance
**1**	**10.92**	**29.51**	**29.51**	**1**	**10.92**	**29.51**	**29.51**	**1**	**10.92**	**29.51**	**29.51**
**2**	**3.22**	**8.70**	**38.21**	**2**	**3.21**	**8.68**	**38.19**	**2**	**3.21**	**8.70**	**38.21**
**3**	**2.07**	**5.60**	**43.81**	**3**	**2.07**	**5.59**	**43.78**	**3**	**2.07**	**5.60**	**43.81**
**4**	**1.58**	**4.28**	**48.09**	**4**	**1.583**	**4.28**	**48.06**	**4**	**1.58**	**4.28**	**48.09**
**5**	**1.48**	**4.00**	**52.09**	**5**	**1.48**	**4.00**	**52.06**	**5**	**1.48**	**4.00**	**52.09**
**6**	**1.24**	**3.34**	**55.41**	**6**	**1.23**	**3.34**	**55.40**	**6**	**1.23**	**3.34**	**55.43**
**7**	**1.18**	**3.19**	**58.62**	**7**	**1.18**	**3.19**	**58.59**	**7**	**1.18**	**3.19**	**58.62**
**8**	**1.09**	**2.94**	**61.56**	**8**	**1.09**	**2.93**	**61.52**	**8**	**1.08**	**2.94**	**61.56**
9	0.96	2.59	64.15	9	0.96	2.59	64.11	9	0.96	2.59	64.15
10	0.92	2.48	66.63	10	0.92	2.47	66.58	10	0.92	2.48	66.63
11	0.89	2.41	69.04	11	0.89	2.41	68.99	11	0.89	2.41	69.04
12	0.83	2.23	71.27	12	0.82	2.23	71.22	12	0.82	2.23	71.27
13	0.76	2.04	73.31	13	0.76	2.04	73.26	13	0.76	2.04	73.31
14	0.74	1.99	75.30	14	0.74	1.99	75.25	14	0.74	1.99	75.30
15	0.68	1.84	77.14	15	0.68	1.84	77.09	15	0.68	1.84	77.14
16	0.65	1.76	78.90	16	0.65	1.75	78.84	16	0.65	1.76	78.90
17	0.59	1.60	80.50	17	0.59	1.60	80.44	17	0.59	1.60	80.50
18	0.59	1.59	82.09	18	0.59	1.59	82.03	18	0.59	1.59	82.09
19	0.56	1.52	83.61	19	0.56	1.52	83.55	19	0.56	1.52	83.61
20	0.54	1.45	85.06	20	0.53	1.44	84.99	20	0.53	1.45	85.06
21	0.51	1.37	86.43	21	0.51	1.37	86.36	21	0.51	1.37	86.43

22	0.50	1.34	87.77	22	0.50	1.34	87.70	22	0.49	1.34	87.77
23	0.47	1.27	89.04	23	0.47	1.26	88.96	23	0.47	1.26	89.03
24	0.44	1.19	90.23	24	0.447	1.19	90.15	24	0.44	1.20	90.23
25	0.43	1.15	91.38	25	0.427	1.14	91.29	25	0.42	1.15	91.38
26	0.37	1.01	92.39	26	0.377	1.00	92.29	26	0.37	1.00	92.38
27	0.36	0.96	93.35	27	0.357	0.96	93.25	27	0.35	0.96	93.34
28	0.34	0.92	94.27	28	0.34	0.91	94.16	28	0.34	0.92	94.26
29	0.33	0.89	95.16	29	0.32	0.87	95.03	29	0.32	0.88	95.14
30	0.31	0.83	95.99	30	0.31	0.83	95.86	30	0.31	0.83	95.97
31	0.27	0.74	96.73	31	0.27	0.74	96.60	31	0.27	0.74	96.71
32	0.26	0.69	97.42	32	0.26	0.7	97.30	32	0.26	0.69	97.40
33	0.25	0.68	98.10	33	0.25	0.68	97.98	33	0.25	0.68	98.08
34	0.22	0.61	98.71	34	0.22	0.63	98.61	34	0.22	0.61	98.69
35	0.20	0.54	99.26	35	0.20	0.57	99.18	35	0.20	0.54	99.23
36	0.19	0.51	99.77	36	0.18	0.56	99.74	36	0.18	0.51	99.74
37	0.09	0.23	100	37	0.09	0.26	100	37	0.09	0.26	100

Extraction method: Principal Component Analysis.

File S1: Cuestionario (Spanish)

El propósito de este estudio es aprender cómo los estudiantes de medicina en Europa son formados sobre la prescripción de antibióticos y el uso apropiado de los mismos (“prescripción adecuada de antibióticos”). Agradecemos mucho que complete la encuesta. Los resultados serán utilizados para sugerir mejoras en los planes de estudio vigentes, y serán publicados en revistas revisadas por pares de nivel nacional e internacional. La encuesta requiere entre cinco y diez minutos para ser completada, por favor intente responder a todas las preguntas. Todas las respuestas son anónimas y serán almacenadas confidencialmente.

1. ¿Qué edad tiene (en años)?

2. ¿Cuál es su sexo?
a. Masculino
b. Femenino

3. ¿Es usted ciudadano del país en el que estudia medicina?
a. Sí.
b. No (por favor, escriba el país del que es ciudadano)

4. ¿Terminará sus estudios de medicina antes de que acabe febrero de 2023?
a. Sí
b. No
C. Otros (por favor, especificar)

5. ¿Cuál es la duración total de su formación médica en tu facultad de medicina?
a. 4 años
b. 5 años
c. 5,5 años/11 semestres
d. 6 años
e. 7 años
f. Otros (por favor, especificar)

6. ¿En qué facultad de medicina está estudiando?

Por favor, tenga en cuenta que esta es la sección más larga de la encuesta.

En esta sección se pregunta respecto a su opinión de si la formación recibida en su facultad de medicina le prepara en los siguientes temas de uso responsable de antibióticos (incluidos todos los cursos, conferencias, prácticas clínicas, capacitación a pié de cama, exámenes y autoevaluación, aprendizaje dirigido) para la correcta práctica clínica cuando comiences como residente de formación sanitaria especializada.

Para cada pregunta, responda "Me siento... (1 = nada preparado, 4 = suficientemente preparado, 7 = muy bien preparado) para...". Si hay preguntas en las que no ha tenido ninguna formación, seleccione "no se recibió formación", independientemente de qué tan bien preparado se sienta sobre este tema.

**Diagnóstico de la infección. Me siento...**	**No se recibió formación**	**1 (nada preparado)**	**2**	**3**	**4 (suficiente mente preparado)**	**5**	**6**	**7 (muy bien preparado)**	**No estoy seguro de cómo me siento**	**No entiendo la pregunta**
**7. Para reconocer los signos clínicos de infección**	O	O	O	O	O	O	O	O	O	O
**8. Para evaluar la severidad clínica de la infección (ej.: utilizando criterios, tales como criterios de shock séptico)**	O	O	O	O	O	O	O	O	O	O
**9. Para usar tests rápidos (ej.: tira de orina, test de estreptococo)**	O	O	O	O	O	O	O	O	O	O
**10. Para interpretar biomarcadores de inflamación (ej.: PCR)**	O	O	O	O	O	O	O	O	O	O
**11. Para interpretar cuando es importante tomar muestras microbiológicas antes de iniciar la antibioterapia**	O	O	O	O	O	O	O	O	O	O
**12. Para interpretar resultados microbiológicos básicos (ej.: hemocultivos, antibiograma con estudio de sensibilidad)**	O	O	O	O	O	O	O	O	O	O

Indicaciones para la no prescripción de antibiótico<br>Me siento…	No se recibió formación	1 (nada preparado)	2	3	4 (suficiente mente preparado)	5	6	7 (muy bien preparado)	No estoy seguro de cómo me siento	No entiendo la pregunta
13. Para identificar situaciones clínicas en las que no prescribir antibióticos	O	O	O	O	O	O	O	O	O	O
14. Para diferenciar entre colonización bacteriana e infección (ej.: bacteriuria asintomática)	O	O	O	O	O	O	O	O	O	O
15. Para diferenciar entre infecciones bacterianas y víricas del tracto respiratorio superior	O	O	O	O	O	O	O	O	O	O

Antibioterapia inicial<br>Me siento…	No se recibió formación	1 (nada preparado)	2	3	4 (suficiente mente preparado)	5	6	7 (muy bien preparadp)	No estoy seguro de cómo me siento	No entiendo la pregunta
16. Para seleccionar antibioterapia empírica inicial basada en los patógenos más comunes y los patrones de resistencia a antibióticos, sin utilizar guías clínicas	O	O	O	O	O	O	O	O	O	O
17. Para decidir la urgencia de la administración del antibiótico en diferentes situaciones (ej.: <1 hora para sepsis severa, no urgente para infecciones crónicas de hueso).	O	O	O	O	O	O	O	O	O	O
18. Para prescribir antibioterapia de acuerdo con las guías nacionales/locales)	O	O	O	O	O	O	O	O	O	O
19. Para evaluar la alergia a los antibióticos (ej.: diferenciar entre anafilaxia e hipersensibilidad)	O	O	O	O	O	O	O	O	O	O
20. Para decidir la duración de antibioterapia más corta adecuada para la infección específica	O	O	O	O	O	O	O	O	O	O
21. Para prescribir usando principios de profilaxis antibiótica ante procedimientos quirúrgicos.	O	O	O	O	O	O	O	O	O	O

Reevaluación de la antibioterapia<br>Me siento…	No se recibió formación	1 (nada preparado)	2	3	4 (suficiente mente preparado)	5	6	7 (muy bien preparado)	No estoy seguro de cómo me siento	No entiendo la pregunta
22. Para revisar la necesidad de continuar o cambiar la antibioterapia tras 48-72 horas, basado en la evolución clínica y los resultados de laboratorio.	O	O	O	O	O	O	O	O	O	O
23. Para evaluar los resultados clínicos y posibles razones para el fracaso del tratamiento antibiótico).	O	O	O	O	O	O	O	O	O	O
24. Para decidir cuando pasar de la antibioterapia intravenosa (IV) a la oral.	O	O	O	O	O	O	O	O	O	O

Calidad del cuidado<br>Me siento…	No se recibió formación	1 (nada preparado)	2	3	4 (suficiente mente preparado)	5	6	7 (muy bien preparado)	No estoy seguro de cómo me siento	No entiendo la pregunta
25. Para medir/revisar el uso de antibiótico en el entorno clínico e interpretar el resultado de dichos estudios	O	O	O	O	O	O	O	O	O	O
26. Para trabajar dentro del equipo multidisciplinar dedicado al manejo de los antibióticos en los hospitales	O	O	O	O	O	O	O	O	O	O

Habilidades comunicativas<br>Me siento…	No se recibió formación	1 (nada preparado)	2	3	4 (suficiente mente preparado)	5	6	7 (muy bien preparado)	No estoy seguro de cómo me siento	No entiendo la pregunta
27. Para medir/revisar el uso de antibiótico en el entorno clínico e interpretar el resultado de dichos estudios	O	O	O	O	O	O	O	O	O	O
28. Para trabajar dentro del equipo multidisciplinar dedicado al manejo de los antibióticos en los hospitales	O	O	O	O	O	O	O	O	O	O

Resistencia antibiótica. Me siento…	No se recibió formación	1 (nada preparado)	2	3	4 (suficiente mente preparado)	5	6	7 (muy bien preparado)	No estoy seguro de cómo me siento	No entiendo la pregunta
29. Para utilizar el conocimiento sobre los mecanismos comunes de resistencia a antibióticos en los patógenos	O	O	O	O	O	O	O	O	O	O
30. Para utilizar el conocimiento sobre la epidemiología de la resistencia bacteriana, incluyendo variaciones locales/regionales	O	O	O	O	O	O	O	O	O	O
31. Para practicar control efectivo de la infección e higiene (para prevenir la diseminación de la bacteria)	O	O	O	O	O	O	O	O	O	O
32. Para usar el conocimiento sobre las consecuencias negativas del uso de los antibióticos (resistencia bacteriana, efectos tóxicos o adversos, coste, infecciones por *Clostridium difficile*).	O	O	O	O	O	O	O	O	O	O

¿Cuáles de los siguientes métodos se han utilizado en su facultad para formar sobre el uso responsable de los antibióticos (los temas del anterior conjunto de preguntas) y cuán útiles fueron?

	El método de enseñanza no ha sido utilizado	No útil en absoluto	Neutral	Útil	Muy Útil	Estoy inseguro	No he entendido la pregunta
33. Conferencias (con <15 personas)	O	O	O	O	O	O	O
34. Enseñanza en grupos reducidos	O	O	O	O	O	O	O
35. Discusión de casos clínicos y viñetas	O	O	O	O	O	O	O
36. Tareas de aprendizaje activo (ej.: lectura de artículos, trabajo en grupo, preparación de presentación oral)	O	O	O	O	O	O	O
37. Aprendizaje electrónico (*E-learning*)	O	O	O	O	O	O	O
38. Role play ou sesións de habilidades comunicarivas tratando con pacientes que	O	O	O	O	O	O	O

**demandan antibioterapia**							
**39. Rotación durante Práctica Clínica en unidades de enfermidades infecciosas (involucrando pacientes).**	O	O	O	O	O	O	O
**40. Rotación en Microbiología**	O	O	O	O	O	O	O
**41. Enseñanza tutorizada o casi turorizada (ej.: clase impartida por otros estudiantes o por médicos recientemente graduados).**	O	O	O	O	O	O	O

42. En general, ¿cree que ha recibido suficiente formación en la facultad de medicina sobre el uso de antibióticos para su práctica futura como residente?

a. Sí

b. No. Siento que tuve suficiente formación sobre el tratamiento antibiótico en general, pero necesito más formación sobre el uso responsable de antibióticos.

c. No. Siento que necesito más formación tanto sobre el tratamiento antibiótico en general como sobre el uso prudente de antibióticos.

d. No estoy seguro

43. ¿Alguno de los exámenes de la facultad de medicina ha incluido preguntas sobre el tratamiento con antibióticos?

a. Sí.

b. No.

C. No estoy seguro.

d. Otros (por favor, especificar)

44. ¿Cómo encontró el lenguaje utilizado en este cuestionario?

a. Pude entender todo o casi todo.

b. Pude entender la mayoría de las preguntas.

c. Muchas preguntas me resultaron difíciles de entender.

d. La mayor parte de la encuesta me resultó difícil de entender.

45. ¿Cómo cree que se puede mejorar la formación sobre el tratamiento antibiótico y el uso prudente de antibióticos?

S2: Questionnaire (English)

The purpose of this study is to understand how medical students in Europe are trained in antibiotic prescribing and appropriate use ("appropriate antibiotic prescribing"). We greatly appreciate your participation in completing this survey. The results will be used to suggest improvements to current curricula and will be published in peer-reviewed journals at both national and international levels. The survey takes approximately five to ten minutes to complete, and we kindly ask that you try to answer all questions. All responses are anonymous and will be stored confidentially.

1. How old are you (in years)?

2. What is your sex?
a. Male
b. Female

3. Are you a citizen in the country where you study medicine?
a. Yes
b. No (please specify your country of citizenship)

4. Will you complete your medical studies before the end of February 2023?
a. Yes
b. No
c. Other (please specify)

5. What is the total duration of your medical training at your medical school?
a. 4 years
b. 5 years
c. 5.5 years / 11 semesters
d. 6 years
e. 7 years
f. Other (please specify)

6. Which medical school are you attending?

Please note that this is the longest section of the survey.

In this section, we ask about your opinion on whether the training provided by your medical school prepares you in the following areas of responsible antibiotic use (including all courses, lectures, clinical practices, bedside training, exams and self-assessment, and directed learning) for effective clinical practice when you start as a medical resident in specialized healthcare training.

For each question, please respond, "I feel... (1 = not prepared at all, 4 = sufficiently prepared, 7 = very well prepared) to..." If there are topics in which you have not received any training, select "no training received," regardless of how well prepared you feel on that topic.

**Infection Diagnosis. I feel...**	**No training received**	**1 (not prepared at all)**	**2**	**3**	**4 (sufficiently prepared)**	**5**	**6**	**7 (very well prepared)**	**I'm not sure how I feel**	**I don't understand the question**
**7. To recognize the clinical signs of infection**	O	O	O	O	O	O	O	O	O	O
**8. To assess the clinical severity of infection (e.g., using criteria, such as septic shock criteria)**	O	O	O	O	O	O	O	O	O	O
**9. To use rapid tests (e.g., urine dipstick, strep test)**	O	O	O	O	O	O	O	O	O	O
**10. To interpret inflammatory biomarkers (e.g., CRP)**	O	O	O	O	O	O	O	O	O	O
**11. To interpret when it is important to collect microbiological samples before starting antibiotic therapy**	O	O	O	O	O	O	O	O	O	O
**12. To interpret basic microbiological results (e.g., blood cultures, antibiogram with antibiotic susceptibility testing)**	O	O	O	O	O	O	O	O	O	O

**Indications for not prescribing antibiotics. I feel...**	**No training received**	**1 (not prepared at all)**	**2**	**3**	**4 (sufficiently prepared)**	**5**	**6**	**7 (very well prepared)**	**I'm not sure how I feel**	**I don't understand the question**
**13. To identify clinical situations in which antibiotics should not be prescribed**	O	O	O	O	O	O	O	O	O	O
**14. To differentiate between bacterial colonization and infection (e.g., asymptomatic bacteriuria)**	O	O	O	O	O	O	O	O	O	O

**15. To differentiate between bacterial and viral upper respiratory tract infections**	O	O	O	O	O	O	O	O	O	O

**Initial Antibiotic Therapy I feel…**	**No training received**	**1 (not prepared at all)**	**2**	**3**	**4 (sufficiently prepared)**	**5**	**6**	**7 (very well prepared)**	**I'm not sure how I feel**	**I don't understand the question**
**16. To select initial empirical antibiotic therapy based on the most common pathogens and antibiotic resistance patterns, without using clinical guidelines**	O	O	O	O	O	O	O	O	O	O
**17. To determine the urgency of antibiotic administration in different situations (e.g., <1 hour for severe sepsis, non-urgent for chronic bone infections)**	O	O	O	O	O	O	O	O	O	O
**18. To prescribe antibiotic therapy in accordance with national/local guidelines**	O	O	O	O	O	O	O	O	O	O
**19. To assess antibiotic allergies (e.g., differentiate between anaphylaxis and hypersensitivity)**	O	O	O	O	O	O	O	O	O	O
**20. To decide on the shortest adequate duration of antibiotic therapy for a specific infection**	O	O	O	O	O	O	O	O	O	O
**21. To prescribe using principles of antibiotic prophylaxis for surgical procedures**	O	O	O	O	O	O	O	O	O	O

**Re-evaluation of Antibiotic Therapy I feel…**	**No training received**	**1 (not prepared at all)**	**2**	**3**	**4 (sufficiently prepared)**	**5**	**6**	**7 (very well prepared)**	**I'm not sure how I feel**	**I don't understand the question**
**22. To review the need to continue or change antibiotic therapy after 48-72 hours, based on clinical progress and laboratory results**	O	O	O	O	O	O	O	O	O	O
**23. To assess clinical outcomes and possible reasons for antibiotic treatment failure**	O	O	O	O	O	O	O	O	O	O
**24. To decide when to switch from intravenous (IV) to oral antibiotic therapy**	O	O	O	O	O	O	O	O	O	O

Quality of Care<br>I feel…	No training received	1 (not prepared at all)	2	3	4 (sufficiently prepared)	5	6	7 (very well prepared)	I'm not sure how I feel	I don't understand the question
25. To measure/review antibiotic use in the clinical setting and interpret the results of such studies	O	O	O	O	O	O	O	O	O	O
26. To work within the multidisciplinary team dedicated to antibiotic management in hospitals	O	O	O	O	O	O	O	O	O	O

Communication Skills<br>I feel…	No training received	1 (not prepared at all)	2	3	4 (sufficiently prepared)	5	6	7 (very well prepared)	I'm not sure how I feel	I don't understand the question
27. To explain the importance of appropriate antibiotic use to patients and their families	O	O	O	O	O	O	O	O	O	O
28. To discuss antibiotic use and resistance issues effectively with healthcare professionals and team members	O	O	O	O	O	O	O	O	O	O

Antibiotic Resistance<br>I feel…	No training received	1 (not prepared at all)	2	3	4 (sufficiently prepared)	5	6	7 (very well prepared)	I'm not sure how I feel	I don't understand the question
29. To apply knowledge of common mechanisms of antibiotic resistance in pathogens	O	O	O	O	O	O	O	O	O	O
30. To use knowledge of the epidemiology of bacterial resistance, including local/regional variations	O	O	O	O	O	O	O	O	O	O
31. To practice effective infection control and hygiene (to prevent bacterial spread)	O	O	O	O	O	O	O	O	O	O
32. To use knowledge about the negative consequences of antibiotic use (bacterial resistance, toxic or adverse effects, cost, *Clostridium difficile* infections)	O	O	O	O	O	O	O	O	O	O

Which of the following methods have been used at your medical school to train you on responsible antibiotic use (the topics from the previous set of questions), and how useful were they?

	The teaching method has not been used	Not useful at all	Neutral	Useful	Very Useful	I'm unsure	I don't understand the question
33. Lectures (with >15 people)	O	O	O	O	O	O	O
34. Small group teaching	O	O	O	O	O	O	O
35. Clinical case discussions and vignettes	O	O	O	O	O	O	O
36. Active learning tasks (e.g., reading articles, group work, preparing oral presentations)	O	O	O	O	O	O	O
37. E-learning	O	O	O	O	O	O	O
38. Role-play or communication skills sessions dealing with patients requesting antibiotic therapy	O	O	O	O	O	O	O
39. Clinical Practice Rotation in Infectious Diseases units (involving patients)	O	O	O	O	O	O	O
40. Microbiology Rotation	O	O	O	O	O	O	O
41. Peer or near-peer teaching (e.g., classes taught by other students or recent medical graduates)	O	O	O	O	O	O	O

42. Overall, do you feel that you have received sufficient training in medical school on antibiotic use for your future practice as a resident?

a. Yes

b. No. I feel I received sufficient training on antibiotic treatment in general, but I need more training on responsible antibiotic use.

c. No. I feel I need more training on both antibiotic treatment in general and prudent antibiotic use.

d. I'm not sure

43. Have any of your medical school exams included questions about antibiotic treatment?

a. Yes

b. No

c. I'm not sure

d. Other (please specify)

44. How did you find the language used in this questionnaire?

a. I could understand everything or almost everything.

b. I could understand most of the questions.

c. Many questions were difficult to understand.

d. Most of the survey was difficult to understand.

45. How do you think training on antibiotic treatment and prudent antibiotic use can be improved?
